# Supplementary material for: Degradable Plasma-Polymerized Poly(Ethylene Glycol)-Like Coating as a Matrix for Food-Packaging Applications
Source: Nanomaterials (Basel). 2023 Oct 16;13(20):2774. doi: 10.3390/nano13202774 (PMC10609115; doi:10.3390/nano13202774)
Supplement: Supplementary file 1 [file nanomaterials-13-02774-s001.zip › nanomaterials-2607014-supplementary.pdf]

Degradable Plasma-Polymerized Poly(ethylene glycol)-Like Coating as a Matrix for Food-Packaging Applications

Maryam Zabihzadeh Khajavi [1,2,*], Anton Nikiforov [2], Maryam Nilkar [2], Frank Devlieghere [1], Peter Ragaert [1]
and Nathalie De Geyter [2]

[1] Research Unit Food Microbiology and Food Preservation, Department of Food Technology, Safety and Health, Ghent University, Coupure Links 653, 9000 Ghent, Belgium; frank.devlieghere@ugent.be (F.D.); peter.ragaert@ugent.be (P.R.)
[2] Research Unit Plasma Technology, Department of Applied Physics, Ghent University, Sint-Pietersnieuwstraat 41, 9000 Ghent, Belgium; anton.nikiforov@ugent.be (A.N.); maryam.nilkar@ugent.be (M.N.); nathalie.degeyter@ugent.be (N.D.G.)
* Correspondence: maryam.zabihzadehkhajavi@ugent.be; Tel.: 032498031287

1. Optimization of atomizer gas flow rate

The atomizer gas flow rate was optimized by coating silicon wafers and examining the stability of the obtained coatings in contact with water. For this purpose, different atomizer gas flow rates (0.6, 0.7, 0.8, 0.9, and 1.0 slm) were applied to conduct depositions at a fixed input power of 175 W and a fixed distance of 2 mm from the edge of the plasma head. After deposition, the samples were immersed in distilled water for up to 72 hours at a constant temperature of 25°C, analyzed by XPS and compared to the chemical composition of the as-deposited coatings. Table S1 indicates the occurring changes in elemental composition of the differently obtained coatings resulting from different immersion times in water. The atomizer gas flow rate of 0.6 slm is considered to be optimal as in this case no silicon can be detected on the coating surface up to 72 hours of water immersion suggesting the deposition of a stable coating. In the case of higher atomizer gas flow rates, silicon originating from the substrate is always observed at the surface suggesting poor coating stability.

Table S1. Changes in surface elemental composition of plasma-polymerized DVE-3 coatings during contact with water as a function of atomizer gas flow rate obtained from XPS

Atomizer flow rate (slm)	Time interval (h)	C (at. %)	O (at. %)	N (at. %)	Si (at. %)
0.6	0	62.9 ± 0.9	35.0 ± 1.1	2.1 ± 0.2	0.0
	1	64.2 ± 2.0	34.2 ± 2.2	1.6 ± 0.2	0.0
	24	62.6 ± 0.9	35.9 ± 0.8	1.5 ± 0.2	0.0
	72	63.3 ± 0.7	35.5 ± 0.8	1.2 ± 0.2	0.0
0.7	0	65.1 ± 1.3	33.4 ± 0.8	1.6 ± 0.5	0.0
	1	63.2 ± 0.5	35.2 ± 0.5	1.6 ± 0.3	0.0
	24	64.2 ± 1.2	33.5 ± 0.5	1.2 ± 0.4	1.1 ± 0.3
	72	58.6 ± 1.9	36.7 ± 1.5	1.4 ± 0.4	3.4 ± 0.3
0.8	0	63.2 ± 0.3	35.1 ± 0.2	1.7 ± 0.1	0.0
	1	57.8 ± 0.6	36.2 ± 0.1	3.0 ± 0.2	3.0 ± 0.5
	24	45.5 ± 0.3	38.9 ± 0.7	5.6 ± 0.3	10.1 ± 0.6
0.9	0	63.6 ± 0.3	35.1 ± 0.3	1.3 ± 0.4	0.0
	1	43.3 ± 0.4	40.7 ± 1.2	5.6 ± 0.8	10.3 ± 0.7
1.0	0	63.3 ± 0.1	35.4 ± 0.2	1.6 ± 0.1	0.0
	1	43.1 ± 0.2	39.5 ± 0.4	5.3 ± 0.4	12.1 ± 0.5
